# An evaluation of speech therapy care in the surrounding area of an interdisciplinary cleft lip and palate tertiary care center

**DOI:** 10.1038/s41598-025-90588-x

**Published:** 2025-02-18

**Authors:** Katharina Schaffrath, Mark Ooms, Simone Seidel, Frank Hölzle, Ali Modabber

**Affiliations:** Department of Oral and Maxillofacial Surgery, University Hospital RWTH Aachen, Pauwelsstraße 30, 52074 Aachen, Germany

**Keywords:** Speech therapy, Cleft, Lip, Palate, Development, Interdisciplinary collaboration, Paediatric research, Paediatric research

## Abstract

The anatomical deformation in cleft patients requires speech therapy to support cleft patients as best as possible. The aim of this study was to evaluate the standard of knowledge of therapists concerning clefts. Furthermore, the study aimed to determine whether there was a difference between therapists with and without treatment experience in cleft patients as well as among therapists with more or less years of general professional experience. We developed a questionnaire about different areas of speech therapy: “General,” “Speech therapy,” “Development opportunities and influences,” and “Interdisciplinary collaboration.” For a total of 50 questions, we used single-, multiple-choice questions and the visual analog scale (VAS). Speech therapists with experience in treating cleft patients (n = 43) felt more confident regarding their knowledge and abilities than therapists without experience (n = 61), especially concerning nonspecialist disciplines and cleft specifications. No difference was found in therapy duration, indications, influences, and potential for development. Professional experience (years) and cleft experience correlate; with more knowledge in the group with more than 8 years of experience. Cleft centers remain first choice for patients’ care thanks to the higher number of patients, daily treatment routine, the direct contact among examiners, and a common treatment concept.

## Introduction

With an incidence of 1:500 to 1:1000 births worldwide, cleft lip and palate is one of the most frequent deformities in newborn children^1^. This condition carries significant anatomical impediments. Therefore, the interdisciplinary care of a patient with cleft lip and palate requires not only the closure of the cleft by a maxillofacial surgeon but also speech therapy to help with swallowing, feeding, and the development of speech. Early intervention by specialists can prevent problems that may develop later^2^. There is a large range of cleft types, which are divided into three groups: (1) lip, (2) lip and palate, and (3) isolated cleft palate^3^. While a cleft of the lip may cause problems with feeding, a cleft palate frequently causes developmental disorders linked to swallowing and speech^4^. Any psychological effects should be addressed. Many cleft children with disorders or delayed development show reduced academic performance and have fewer social and professional opportunities in adulthood^5^.

In 50% of children with cleft palate, speech therapy is required to train newly built or underdeveloped anatomical structures^6^. Non-cleft patients show a median nasality in vocals of 35.9%; in typical test sentences, they show a median nasality of 24.9%^7^. Patients with cleft palate show a significantly higher nasality than patients with isolated cleft lip^8^. This may be due to organic deficits, functional disorders, or both^7^. Despite the use of speech therapy, many children still face difficulties, such as consonant articulation and open rhinophonia, which is an indication of the need for further operative correction. This type of correction must always be suggested by surgeons and speech therapists together^9^. In some cases, a simultaneous operation in two areas is beneficial for the patient; for example, velopharyngoplasty and tonsillectomy. With this approach, there is shorter narcosis. Furthermore, there is no significant difference between the quality of spontaneous speech and intraoperative and postoperative complications, so the patient seems to benefit from a simultaneous operation^10^. In this case, it is important for both the patient and the surgeon to discuss preoperative and postoperative expectations (e.g., anatomical issues and speech difficulties linked to velopharyngeal insufficiency) with speech therapists. The expected success of speech therapy must always be assessed by a speech therapist. The age at which the operation is performed is essential for the patient’s development. For effective social and psychological rehabilitation, it should be performed before school age^9^. The decision is related to the grade of nasality and the velopharyngeal insufficiency, which persists in 15–30% of children with cleft palate^11^. Although lips seem to have a lower influence on speech development, some abnormal learned neuromotor patterns could make speech therapy necessary^12^. Treatment is multidisciplinary, lasts up to adulthood, and switches from one discipline to another at different ages.

Due to the importance of patient support, this study aimed to evaluate the standard of knowledge about the topic of cleft lip and palate in speech therapists. The hypothesis was that therapists with many years of professional experience and treatment experience with cleft patients feel more confident regarding their therapeutic practices and assessments. Based on this assumption, treatment in interdisciplinary cleft centers with cleft-specific speech therapists should be preferred.

## Materials and methods

### Development of the questionnaire

After institutional approval by the committee of RWTH Aachen University (EK23-277), three surgeons and a speech therapist with experience treating cleft patients developed the questionnaire (Supplementary Material). These health-care professionals belonged to the Cleft Center of RWTH Aachen University Hospital. To evaluate and improve the content and type of questions we asked three students of the school for speech therapy of the RWTH Aachen University and three experienced speech therapists dedicated to the cleft center to answer the questionnaire. No criticism was expressed. The questionnaire addressed four topics, which were given the following labels: “General,” “Speech therapy,” “Development opportunities and influences,” and “Interdisciplinary collaboration.” It consisted of 50 questions. The first topic was explored via 6 questions on treatment experience and the subjective assessment of confidence in treating cleft patients. The second topic relied on 14 questions about speech therapy, particularly special diagnostic and therapeutic measurements in cleft therapy. The third topic (16 questions) dealt with the opportunities for development compared to non-cleft patients and influences such as therapy motivation, social components, and type of cleft. The last 14 questions asked about interdisciplinary collaborations between the most important disciplines involved in this field, including surgery, otolaryngology, orthodontics, and speech therapy. Information concerning age, sex, professional experience in years, qualifications (no degree, apprenticeship, bachelor, master, and diploma), and special or additional qualifications was also collected. All the participants were instructed to answer the questions to the best of their knowledge and beliefs regardless of whether they had experience treating cleft patients. The questionnaire included single- and multiple-choice questions but no open questions. For 36 questions, we used the VAS to evaluate subjective assessments following Lee et al.^13^. Small values meant little approval; high values meant high approval.

### Data collection and statistical analysis

The questionnaire was sent to 520 speech therapists chosen via the internet in the region of Aachen, North Rhine-Westphalia, Germany, within a radius of 60 km from the center that delivers cleft-related tertiary care at Aachen University Hospital. The statistical analysis focused on the general educational status of the therapists, the comparison between therapists with and without cleft-related treatment experience, and their years of practice.

In total, 115 anonymized questionnaires were received. Of these, 8 were excluded due to missing degrees at the time of the survey. Three more were excluded because of missing answers, except for the baseline information. Thus, 104 questionnaires were included.

The questionnaire was divided into nine categories, each with a specific focus. In questions 1–3, the participants provided baseline information about their age, sex, qualifications, and experience of treating cleft patients. Questions 4–6 dealt with the subjective assessment of confidence in treating cleft patients. Questions 7–14 explored the topics of diagnosis and therapy, whereas questions 15 and 16 focused on the age at which therapy should be performed. Questions 17 and 18 looked at the indications for speech therapy in cleft patients, and questions 19 and 20 investigated the special symptomatology of such patients. The influence of exogenous factors was addressed in questions 21–26, and the potential for development in comparison to non-cleft individuals was examined in questions 27–36. Finally, the issue of interdisciplinary collaborations was dealt with in questions 37–41 and that of nonspecialist disciplines in questions 42–50.

Single questions were excluded from the evaluation if there was no answer, no clearly evaluable answer, or an answer that did not match the question (e.g., if an age was given when asked for a period). Questions 12 and 13 were excluded from the evaluation because they were based on question 11, which most participants answered by stating a lack of knowledge.

We sought to verify whether there was any difference in knowledge between cleft-experienced therapists (CETs, n = 43) and non-cleft-experienced therapists (NCETs, n = 61) and whether there was any difference depending on general professional experience. The participants were divided into two groups based on their median experience, which was 9 years. Participants with little experience (LE, up to 8 years, n = 53) were assigned to group 1, while those with extensive experience (EE, over 8 years, n = 51) were assigned to group 2. The hypothesis was that therapists with more professional experience and more experience treating cleft patients possessed more knowledge of the problem (“ARM1”).

To address potential bias concerning the correlation between special cleft experience and years of general experience we considered a second statistical arm dividing the participants into four groups (CET EE, n = 31), (CET LE, n = 12), (NCET EE, n = 22) and (NCET LE, n = 39)—in the following describes as “ARM2”.

The statistical analysis was carried out with Microsoft Excel, IBM SPSS Statistics v. 28 (IBM, New York, USA), and Graph Pad Prism 10. Levels of significance were evaluated with the chi-square test for baseline data, the Fisher-Freeman-Halton test for categorical data, and the Mann–Whitney test for metric data. *P* values below 0.05 were considered to be statistically significant.

## Results

This crossover study contained 104 valid questionnaires, each obtained from one speech therapist (4 males and 100 females). Concerning qualifications, 30 therapists had done an apprenticeship, and 74 had completed a degree (bachelor = 51, master = 12, and diploma = 11). Of the participants, 31 had special qualifications in addition to their degrees; these were heterogeneous and not specifically related to the care of cleft patients (e.g., therapy of dysphagia, dyscalculia, dyslexia, facial palsy, and health-care management).

The questionnaires included 43 CETs and 61 NCETs. The CET group also provided information concerning how long they had treated cleft patients and how many patients they had treated in their careers. All the therapists (except five) had worked with up to 10 patients in their careers. The remaining five had treated more patients (two participants had worked with between 10 and 20 patients; one therapist had treated between 20 and 50 patients, and two had worked with more than 50 patients). Seventeen therapists had treated cleft patients for over 10 years; five answered that they had worked with them for between 5 and 10 years. Twelve participants had treated cleft patients for 1–5 years, and eight had very little experience (i.e., less than 1 year).

A correlation was found between professional experience and cleft experience, as the EE therapists had also more experience of treating cleft patients both in terms of years and number of patients. Compared to the NCET group, the CET group included a higher share of EE therapists (ɸ = 0.309).

### Treatment confidence

**Fig. 1:**
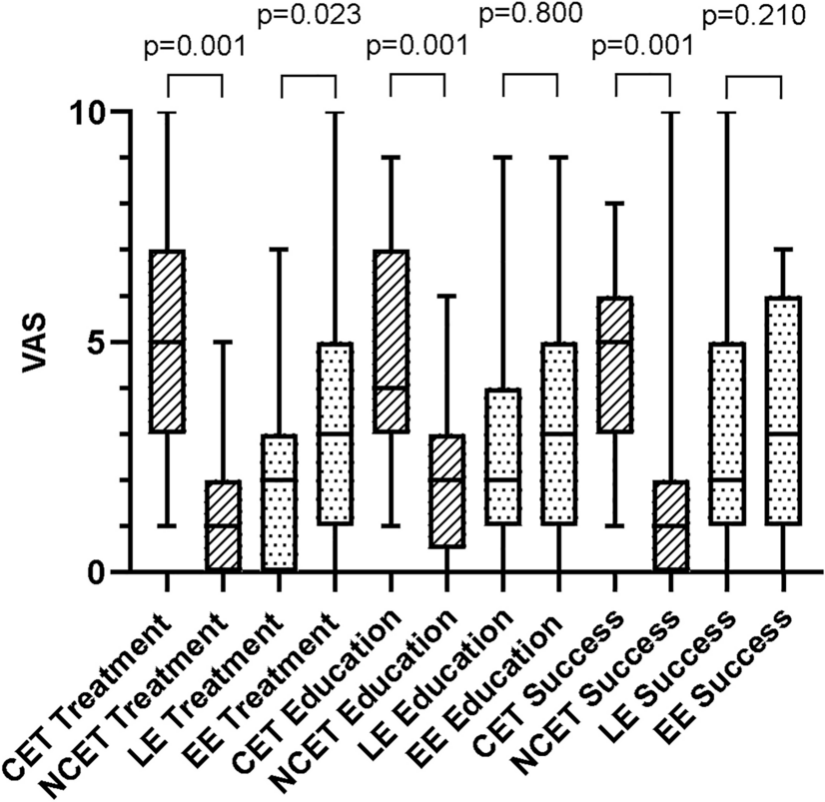
Safety of therapists concerning their treatment, education and expectation of therapy success with cleft patients. CET: cleft experience therapists, NCET: no cleft experience therapists, LE: less experience (up to 8 years), EE: extensive experience (over 8 years).

The VAS clearly showed that CETs felt more confident about the treatment they administered (5 [4]) and its expected outcome (5 [3]) compared to the NCETs. The latter only reached a score of 1 (2) regarding the treatment and its outcome (Table 1). A similar pattern was found for the issue of professional experience. EE therapists felt significantly more confident than LE ones (Fig. 1). No significant difference was found between these two groups with regard to education, but there was a difference between CETs and NCETs—the former felt better educated than the latter (Fig. 1). This result may be caused by the gap between advanced training and basic training. Participants’ confidence regarding the therapy’s success seemed to correlate with their experience of treating cleft patients (Fig. 1). This could be confirmed by ARM2 as it shows a differences between CET and NCET groups but not between experience groups (Table 2).

**Table 1:** Subjective evaluation of speech therapists concerning treatment, influences, patients potential and interdisciplinarity via VAS (ARM1).

How safe do you feel in… Median (i.r.)	CET	NCET	*p*-value	LE	EE	*p*-value
Treatment of cleft patients	5 (4)	1 (2)	0.001	2 (3)	3 (4)	0.023
Education about cleft patients	4 (4)	2 (3)	0.001	3 (3)	3 (4)	0.800
Expectation of therapy success	5 (3)	1 (2)	0.001	2 (4)	3 (5)	0.206
How familiar are you in…						
Castillo-Morales therapy	3 (5)	1 (3)	0.001	1 (3)	3 (6)	0.010
NAM therapy	0 (0)	0 (0)	0.071	0 (0)	0 (0)	0.153
Feeding plates	2 (5)	0 (2)	0.001	0 (3)	2 (4)	0.084
Influence on development of…						
Speech therapy	8 (2)	8 (3)	0.627	7 (3)	8 (3)	0.269
Patient’s motivation	8 (2)	8 (3)	0.443	8 (3)	9 (3)	0.443
Parents	8 (2)	8.5 (3)	0.620	8 (2)	9 (2)	0.127
Social environment	7 (4)	7 (2)	0.717	7 (2)	7 (4)	0.414
Surgery	9 (1)	9 (2)	0.051	9 (2)	9 (2)	0.587
Cleft width	7.5 (2)	7.5 (2)	0.844	7 (1)	8 (3)	0.179
Potential of patients in…						
General development	4 (2)	4 (2)	0.675	4 (2)	4 (2)	0.847
Phonetics	3 (2)	3 (2)	0.643	3 (1)	3 (1)	0.891
Phonology	4 (2)	4 (2)	0.255	4 (2)	4 (2)	0.720
Understanding of speech	5 (0)	5 (0)	0.165	5 (0)	5 (0)	0.880
Production of speech	4 (2)	3 (2)	0.118	3 (1)	4 (2)	0.188
Morphology and syntax	5 (0)	5 (0)	0.292	5 (0)	5 (0)	0.866
Orofacial functions	2 (1)	2 (2)	0.311	2 (2)	2 (2)	0.894
Voice	3 (1)	3 (2)	0.874	3 (2)	3 (2)	0.588
Resonance	3 (1)	2 (1)	0.183	3 (1)	3 (1)	0.890
Oral fluency	5 (0)	5 (1)	0.007	5 (1)	5 (1)	0.527
How important is…						
Surgery information	8 (2)	9 (2)	0.016	8 (3)	8 (3)	0.305
Collaboration with surgeons	9 (3)	9 (3)	0.847	8 (3)	9 (3)	0.240
Collaboration with other disciplines	9 (2)	9 (2)	0.745	9 (2)	9 (2)	0.301
Cleft Center health care	8 (3)	8 (4)	0.923	8 (4)	8 (3)	0.307
Cleft Center speech therapy	7 (4)	7 (5)	0.722	7 (4)	7 (4)	0.720
What do you know about…?						
Anatomy of clefts	6 (2)	2 (4)	0.001	3 (4)	4 (5)	0.092
ENT pathology	6 (3)	2 (4)	0.001	4 (3)	5 (4)	0.173
Orthodontics pathology	5 (3)	1.5 (3)	0.001	2 (4)	3 (4)	0.514
Palate dehiscences	3 (4)	0 (1)	0.001	0 (2)	2 (4)	0.002
Experience with dehiscences	1 (3)	0 (0)	0.001	0 (0)	0 (2)	0.018

Data described as median (with interquartile range) for each question separately for groups (CET vs. NCET; LE vs. EE); *p*-value corresponding to testing for differences between groups with Mann Whitney test; abbreviations: CET: cleft experience therapists, NCET: no cleft experience therapists, LE: less experience (up to 8 years), EE: extensive experience (over 8 years).

**Table 2:** Subjective evaluation of speech therapists concerning treatment, influences, patients potential and interdisciplinarity via VAS (ARM2).

How safe do you feel in… Median (i.r.)	CET EE	CET LE	NCET EE	NCET LE	*p* value
Treatment of cleft patients	5 (4)	3 (3)	1 (2)	1 (2)	0.001
Education about cleft patients	4 (5)	4 (4)	1.5 (2)	2 (2)	0.001
Expectation of therapy success	5 (3)	5.5 (4)	1 (2)	1 (3)	0.001
How familiar are you in…					
Castillo-Morales therapy	4 (5)	3 (3)	2 (4)	1 (2)	0.001
NAM therapy	0 (0)	0 (0)	0 (0)	0 (0)	0.086
Feeding plates	2 (5)	1 (5)	0 (2)	0 (2)	0.001
Influence on development of…					
Speech therapy	8 (3)	7 (3)	8 (4)	7 (2)	0.371
Patient’s motivation	8 (3)	7.5 (3)	9 (4)	8 (3)	0.410
Parents	8 (3)	8 (2)	9 (3)	8 (3)	0.389
Social environment	7 (4)	7 (3)	7 (3)	7 (2)	0.925
Surgery	9 (2)	8 (1)	10 (2)	9 (2)	0.160
Cleft width	7 (2)	8 (2)	8 (3)	7 (1)	0.432
Potential of patients in…					
General development	4 (1)	3.5 (1)	3 (3)	4 (2)	0.351
Phonetics	2 (2)	3 (2)	3 (2)	3 (1)	0.689
Phonology	4 (2)	3.5 (2)	4 (2)	4 (2)	0.667
Understanding of speech	5 (0)	5 (0)	5 (0)	5 (0)	0.309
Production of speech	4 (2)	4 (1)	4 (2)	3 (1)	0.300
Morphology and Syntax	5 (0)	5 (0)	5 (0)	5 (0)	0.687
Orofacial functions	2 (2)	2 (1)	2 (2)	2 (2)	0.753
Voice	3 (2)	3 (1)	3 (2)	3 (2)	0.968
Resonance	3 (1)	3 (2)	2 (1)	3 (1)	0.232
Oral fluency	5 (0)	5 (0)	5 (1)	5 (1)	0.042
How important is…					
Surgery Information	8 (3)	7 (2)	9 (3)	9 (2)	0.100
Collaboration with surgeons	9 (3)	7 (3)	9.5 (3)	9 (3)	0.444
Collaboration with other disciplines	9 (2)	8 (2)	10 (3)	9 (2)	0.288
Cleft Center health care	8 (3)	7 (4)	9.5 (3)	8 (3)	0.358
Cleft Center speech therapy	7 (3)	6 (3)	6.5 (6)	7.5 (4)	0.279
What do you know about…?					
Anatomy of clefts	7 (2)	5 (3)	2 (3)	3 (4)	0.001
ENT pathology	6 (2)	5 (3)	2 (2)	3 (4)	0.001
Orthodontics pathology	5 (3)	5 (3)	1.5 (2)	1.5 (2)	0.001
Palate dehiscences	3 (4)	2 (5)	1 (2)	0 (1)	0.001
Experience with dehiscences	1 (3)	1.5 (3)	0 (0)	0 (0)	0.001

Data described as median (with interquartile range) for each question separately for groups (CET EE vs. CET LE vs. NCET EE vs. NCET LE ); *p*-value corresponding to testing for differences between groups with Mann Whitney test; abbreviations: CET EE: cleft experience therapists and extensive experience (over 8 years), CET LE: cleft experience therapists and limited experience (up to 8 years), NCET EE: no cleft experience therapists and extensive experience (over 8 years), NCET LE: no cleft experience therapists and limited experience (up to 8 years).

### Diagnosis and therapy

The participants were asked about gadgets used in cleft therapy, such as the Castillo-Morales plate, nasoalveolar molding, and feeding plates. Unsurprisingly, CETs and EE therapists knew more about feeding plates and Castillo-Morales plates (Table 1), but there seemed to be almost no knowledge of nasoalveolar molding and the corresponding plate in the entire sample (Table 3). The participants were also asked about special diagnostic and therapeutic measures and methods. As expected, the CET group was significantly more knowledgeable about diagnostic methods in cleft patients, especially in the fields of orofacial diagnosis (CETs = 88.4%, NCETs = 49.3%, *p* = 0.047), basic diagnostic methods (CETs = 48.8%, NCETs = 17.3%, *p* = 0.002), and the mirror investigation (CETs = 67.4%, NCETs = 28.8%, *p* = 0.001) for treating nasality. Knowledge of advice on drinking and breastfeeding was also more widespread in CETs and EE therapists (CETs = 46.7%, NCETs = 20%, *p* = 0.008; LE therapists = 33.3%, EE therapists = 42.6%, *p* = 0.046). When considering ARM2 there is a significant difference in diagnostic and therapeutic measurements as the NCET EE group knows and uses less of the requested methods (Table 4).

**Table 3:** Knowledge of speech therapists about intraoral therapy concepts (ARM1).

I don’t know… Absolute (% of group)	CET	NCET	*p*-value	LE	EE	*p*-value
Castillo-Morales therapy	1 (4.9%)	3 (4.9%)	0.641	3 (5.7%)	1 (2%)	0.618
NAM therapy	29 (67.4%)	43 (70.%)	0.830	42 (79.2%)	30 (58.8%)	0.033
Feeding plates	6 (14%)	16 (26.2%)	0.151	15 (28.3%)	7 (13.7%)	0.093

Data described as numbers (with percentage) for each question separately for groups (CET vs. NCET; LE vs. EE); *p*-value corresponding to testing for differences between groups with chi squared test or Fisher Freeman Halton test; abbreviations: CET: cleft experience therapists, NCET: no cleft experience therapists, LE: less experience (up to 8 years), EE: extensive experience (over 8 years).

**Table 4:** Knowledge of speech therapists about diagnostic and therapeutic methods (AMR2).

I don’t know any of the requested … Absolute (% of group)	CET EE	CET LE	NCET EE	NCET LE	*p*-value
Use of diagnostic methods	0 (0%)	0 (0%)	4 (25%)	2 (5.6%)	0.012
Use of therapeutic methods	0 (0%)	0 (0%)	3 (17.6%)	1 (3%)	0.028
Gadget-ralated methods	0 (0%)	0 (0%)	3 (18.8%)	3 (8.3%)	0.065

Data described as numbers (with percentage) for each question separately for (CET EE vs. CET LE vs. NCET EE vs. NCET LE); *p*-value corresponding to testing for differences between groups with chi squared test or Fisher Freeman Halton test; abbreviations: CET EE: cleft experience therapists and extensive experience (over 8 years), CET LE: cleft experience therapists and limited experience (up to 8 years), NCET EE: no cleft experience therapists and extensive experience (over 8 years), NCET LE: no cleft experience therapists and limited experience (up to 8 years).

### Beginning of speech therapy

All the participants agreed that the diagnosis of the problem and the start of the therapy should happen as soon as possible after birth.

### Indications for speech therapy

The participants were asked about cleft-specific indications and the frequency of speech therapy. No significant differences between the groups were found in this domain. All the therapists agreed that problems with eating and drinking (98%), speech development (91%), orofacial status (97%), hearing (57%), and expression (56%) were indications for speech therapy, while problems concerning fine (26%) and gross (17%) motor skills and reception (41%) did not seem to constitute indications. The frequency of the therapy depended on the patient’s development status (86.6%), age (67%), and compliance (75.3%), as well as on the parents’ compliance (64.9%) and the type (64.9%) and width (53.6%) of the cleft. Sex (3.1%) and the social environment (16.5%) did not have an impact on the frequency of speech therapy.

### Special symptomatology in cleft patients

All the participants agreed that problems in orofacial status (87.5%), phonetics (84.4%), and voice (72.9%) are the most frequent symptoms in cleft patients, while the capability to produce speech also seems to be reduced (61.5%). Problems related to phonology (29.2%), understanding speech (5.2%), oral fluency (5.2%), and morphology and syntax (1%) were considered rare.

### Influence of exogenous factors

No significant differences were found in this domain between CETs and NCETs as well as between LE therapists and EE ones. All the participants thought that factors such as speech therapy, patient motivation, parents, social environment, surgery, and the width of the cleft have a significant influence on the development of the patient’s speech (Table 1). This could be supported by ARM2 (Table 2).

### Potential for development

The participants were asked about the potential for the development of cleft patients compared to that of non-cleft patients in terms of the general development of speech, phonetics, phonology, understanding of speech, production of speech, morphology and syntax, orofacial functions, voice, resonance, and oral fluency. There was no significant difference between the groups. However, according to the respondents, cleft patients have lower potential concerning phonetics, phonology, production of speech, orofacial functions, voice, and resonance. The participants believed that cleft patients have the same potential with regard to the understanding of speech, morphology, syntax, and oral fluency. Values below five indicate lower potential, while values above five signal higher potential compared to non-cleft patients (Table 1). This could be supported by ARM2 (Table 2).

### Interdisciplinary collaboration

In general, the respondents showed considerable interest in additional information from surgeons and nonspecialist disciplines, such as otolaryngology and orthodontics. Only the interest in surgeon support was significantly higher in the NCET group compared to the CET group. All the participants thought that the care provided in an interdisciplinary cleft center is more important than speech therapy itself for the patient’s general development (Table 1).

The respondents were asked about what kind of information they would like to receive from surgeons. Except for information regarding the morphology of the soft palate (LE therapists = 48.1%, EE therapists = 70%, *p* = 0.028), no significant differences were found. The participants replied that almost all kinds of information are helpful, including the type of cleft (94.1%), dehiscence of the palate (87.3%), localization and width of the cleft (86.3%), age at which to operate (81.4%), assessment of surgical possibilities (77.5%), and nonsurgical therapy (73.5%). Only operative techniques (46.1%) seemed to be of less interest. The respondents felt that information about orofacial status (93%), phonetics (83%), and voice and resonance (80%) are important for surgeons.

### Nonspecialist disciplines

**Fig. 2:**
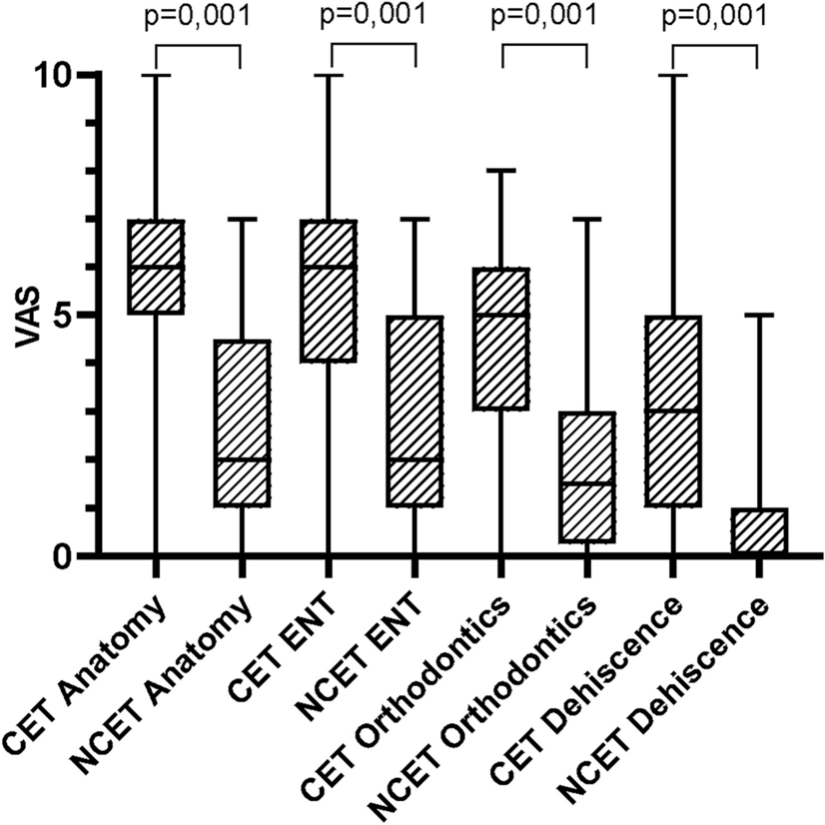
Knowledge of therapists about other disciplines. CET: cleft experience therapists, NCET: no cleft experience therapists.

In this domain, we asked about the anatomy of the palate, the resulting ENT pathologies and orthodontics, the problem of remaining dehiscence of the palate (e.g., after the failure of surgery), and participants’ experiences with these dehiscences (Table 1). The results showed that CETs had considerably more knowledge of these issues than NCETs, while there was almost no difference between LE and EE therapists (Fig. 2). When asked about what types of clefts cause ENT (n = 23) and orthodontic (n = 30) problems, very few evaluable replies were given, which may have been caused by a lack of insight into other disciplines.

### What has more impact—special cleft experience or years of general experience?

It has been proven that CET group performs better than NCET therapists as well as EE perform better than LE therapists (Table 1). This could be supported by ARM 2, by dividing into the groups CET EE, CET LE, NCET EE and NCET LE (Table 2). There remains the question if cleft experience or years of general experience have more impact on therapists’ knowledge (Questions Q42, Q43, Q45, Q47, Q48: What do you know about…?) and self-confidence (Questions Q4, Q5, Q6: How safe do you feel in…?). Comparing CET EE and CET LE whose experience in cleft therapy does not differ significantly, the following can be shown here: there is no significant difference concerning the mentioned questions (Q4: *p* = 0.205; Q5: *p* = 0.712; Q6: *p* = 0.583; Q42: *p* = 0.212; Q43: *p* = 0.118; Q45 *p* = 702; Q47: p = 0.388; Q48: *p* = 0.854). That leads us to the point that cleft experience has more impact on knowledge and self-confidence than general years of experience and that knowledge about nonspecialist disciplines correlate with treatment and expected therapy success.

## Discussion

After sending out 520 questionnaires, we received 115 completed ones (22.1% response rate); 104 were deemed valid. In the final sample, 43 participants had experience treating clefts, while 62 did not have this experience, which represents an adequate distribution. The respondents were self-recruited and chosen via the internet. Every practice that was found online was sent as many questionnaires as the number of therapists working there. However, it can be assumed that older therapists do not have an online presence; hence, we might have missed professionals with significant experience. Speech therapists often work together as a community. So, it is conceivable that they benefit from each other’s knowledge without having extensive experience or education individually. Additionally, there might have been a selection based on interest in the topic and the study, and fewer answers might have come from practices without affected patients. Therefore, one limitation of this survey is the number and reachability of participants, which is a problem that affects all surveys. Another limitation might be that we used a survey for regional cleft care that is not validated.

There is a potential bias due to the correlation between the years of experience and specific cleft treatment experience as there are more experienced therapists in the CET group (ɸ = 0.309). To process this bias, we inserted the second arm of evaluation (ARM2) dividing into four groups. Because of the unequal group sizes, we decided to focus on ARM1 but used ARM2 as a supportive tool which confirmed the results of ARM1 in almost all aspects. In general, there was only minor deviation in the level of significance which supported the thesis of small bias in ARM1. However, it can be emphasized through ARM2 that NCET EE has significant less knowledge on diagnostic and therapeutic methods, even as NCET LE (Table 4). This might be a hint that the education concerning special (also unspecific) methods could have enhanced over the last years, so therapists are better prepared. Younger unexperienced therapists with and without specific cleft experience are more familiar with certain methods—weather it is achieved through speech therapy school or self-motivated. However, this could be explored in further studies.

There is a lack of information in the literature about speech therapy in cleft centers, especially regarding the objective and subjective abilities of therapists. Hence, this study aimed to evaluate therapists’ subjective abilities by exploring their levels of knowledge of cleft lip and palate. Another limitation of this study is that there can be a discrepancy between subjective abilities and objective treatment skills. The treatment delivered in interdisciplinary cleft centers requires legitimation as it involves long journeys and significant efforts for many patients. Many children need speech therapy once a week, and the center’s location causes logistic problems for parents and children because visiting the clinic so often is impossible. This is why treatment opportunities close to the patient’s home are an important factor for successful care.

Cleft patients typically exhibit passive patterns of articulation, which are a product of structural abnormality or dysfunction, and active patterns of articulation, which result from replacing methods^14^. Thus, it is not surprising that speech therapists are interested in when operations can be performed, as we found out through question 37 (“What kind of information from surgeons is important for you as a speech therapist?”). Passive patterns may change after surgery, but not active ones. This is when speech therapy is needed.

Regarding interdisciplinary collaborations with ENT doctors, we should note that there is a high prevalence of middle-ear dysfunction in patients with cleft palate^15^, even after palatal repair^16^. If patients have limited hearing because of frequent otitis media, the success of speech therapy is reduced^17^. The time at which tubes are inserted in case of otitis media directly impacts the beginning of speech therapy and its expected outcome, so either knowledge or interdisciplinary collaboration is needed here. Only a very small number (n = 23) of participants answered question 43 (“How is your knowledge of ENT medicine?”). Those who did answer showed that this knowledge was clearly significant; the CET group was considerably more informed (6 [3]) about ENT pathologies than the NCET group (2 [4]) (Fig. 2). The question remains as to whether this knowledge can be improved in cleft-center therapists who stay in direct contact with ENT doctors.

The same case can be made for orthodontic treatment. When moving palatal segments, passive patterns and, as a consequence, active ones may change. The exchange of information about planned treatment between speech therapists and orthodontists can benefit patients. As shown by the part of the questionnaire on interdisciplinary collaborations, CETs knew significantly more (5 [3]) about orthodontics than NCETs (1.5 [3]) (Fig. 2). Although maxillary expansion may improve nasal breathing and masticatory function as well as enhance aesthetics^18^, it initially enlarges palatal dehiscence and velopharyngeal insufficiency. Because of the movement involved in this intervention, the nasality of speech and the room for the tongue to build sounds may change in the phase of intense orthodontic treatment. In alveolar cleft patients, the success of speech therapy depends on factors such as the shift of the segments of the upper jaw. Hence, the order of different medical interventions and their progress are important elements in speech therapy.

In contrast to surgery, which is responsible for physical alterations and the correction of passive patterns, speech therapy is indicated for the production of compensatory articulation (i.e., the correction of active patterns), whereby articulation placement is changed in response to the abnormal structure^19^. In general, speech therapists distinguish between the understanding of speech, the production of speech, phonetics, phonology, morphology/syntax, orofacial status, voice, resonance, and oral fluency. Frequent problems concerning the development of speech in cleft palate patients are resonance, especially hyper and hypo nasality, and nasal emission, which is a measure of consonant production^20^ due to the special anatomy present in such patients. A major difficulty when treating cleft patients is that the condition changes due to surgery, orthodontics, and ENT pathologies. In non-cleft patients, the therapist can rely on their skills without having to worry about these aggravating factors. Dealing with these factors can be considerably easier in a cleft center where a specific treatment plan involving all relevant disciplines can be devised. This may be one of the reasons why the respondents thought that treatment in an interdisciplinary cleft center is meaningful, regardless of whether they were CETs (8 [3]) or NCETs (8 [4]). CETs answered the question “How confident do you feel treating cleft patients?” with a middle value of 5 (4). The other groups felt even less confident (NCETs = 1 [2]; LE therapists = 2 [3]; EE therapists = 3 [4]) (Fig. 1). One reason for this result could be the lack of cleft-specific basic training (“How well-trained do you feel in cleft treatment?”; CETs = 4 [4], NCETs = 2 [3], LE therapists = 3 [3], EE therapists = 3 [4]). Only the CET group replied to this question positively, which may relate to the possibility that the CETs self-educated in this field.

Even when adequate speech therapy can be accessed away from a cleft center, early treatment is still missing. After their child’s birth, parents often feel overcome by the new situation. Mothers especially feel fear, despair, grief, and guilt after the diagnosis^21^. Many parents know nothing about the cleft before birth. A prenatal diagnosis is still challenging and depends on the experience of the sonographer^22^. The recognition of a soft-tissue cleft is still rare even with experienced examiners^23^, and the visualization of palatal landmarks via sonography depends on gestational ages^24^. Because of these uncertainties, practical and psychological support for parents should begin on day one with the impression and production of a feeding plate (maxillofacial surgeon or dentist) and instruction concerning swallowing and breastfeeding (speech therapist). Subsequent nasoalveolar molding (if indicated) highlights the interdisciplinary nature of the problem because it requires the work of all relevant disciplines applied to one plate for swallowing, feeding, moving soft tissues (nose and lip), and reducing the size of the cleft for future surgery^25^.

The study’s results show that treatment via cleft-specific therapists may be more precise and adequate for patients’ needs. However, question 3 revealed that the respondents with this kind of experience had only worked on a small number of cases; 38 out of 43 participants (88.4%) had seen fewer than 10 cleft patients in their careers. Therefore, future studies should seek to verify whether there is any difference between the subjective abilities of speech therapists working in cleft centers and those of therapists working elsewhere, as there seems to be an effect on the level of experience and number of cases. Scholars should also evaluate whether objective treatment skills correlate with subjective abilities.

## Conclusions

This crossover study shows that speech therapists with experience in the treatment of cleft patients feel more confident regarding their knowledge and treatment capabilities than therapists without this experience. In particular, the former seemed to possess better knowledge of nonspecialist disciplines and cleft specifications, while no difference was found concerning issues such as age at which therapy should be performed, indications, influences, and potential for development. The chance to discuss interdisciplinary problems with colleagues possessing the same levels of information and education is of great benefit to all those involved in this field—maxillofacial surgeons, speech therapists, ENT doctors, and orthodontists. Thanks to their high number of patients and daily treatment routines, interdisciplinary cleft centers remain the first choice for cleft-patient care.

## Supplementary Information

Supplementary Information 1.

Supplementary Information 2.

## Data Availability

The datasets used and analysed during the current study are available from the corresponding author on reasonable request.

## Abbreviations

CET: Cleft experience therapists
NCET: No cleft experience therapists
LE: Therapists with less experience (up to 8 years)
EE: Therapists with extensive experience (over 8 years)
CET EE: Cleft experience therapists and extensive experience (over 8 years)
CET LE: Cleft experience therapists and limited experience (up to 8 years)
NCET EE: No cleft experience therapists and extensive experience (over 8 years)
NCET LE: No cleft experience therapists and limited experience (up to 8 years)
