# Supplementary material for: An evaluation of speech therapy care in the surrounding area of an interdisciplinary cleft lip and palate tertiary care center
Source: Sci Rep. 2025 Feb 18;15:5841. doi: 10.1038/s41598-025-90588-x (PMC11836121; doi:10.1038/s41598-025-90588-x)
Supplement: Supplementary file 1 — Supplementary Information 1. [file 41598_2025_90588_MOESM1_ESM.pdf]

Breakdown concerning the number of responses to each question answered

Question		processed	answers total	ARM 1				ARM 2			
				CET	NCET	EE	LE	CET EE	CET LE	NCET EE	NCET LE
1	Do you treat or have you ever treated patients with cleft lip and palate?	baseline	104	43	61	51	53	31	12	22	39
2	If so, how long have you been treating patients with cleft lip and palate?	baseline	43	43	0	29	14	31	12	0	0
3	How many patients with cleft lip and palate have you already treated?	baseline	43	43	0	29	14	31	12	0	0
4	How confident do you feel in treating patients with cleft lip and palate?	table 1.1 table 2.1	104	43	61	51	53	31	12	22	39
5	How well educated do you feel in dealing with cleft patients?	table 1.1 table 2.1	104	43	61	51	53	31	12	22	39
6	How confident do you feel about the expected therapeutic outcome of cleft patients?	table 1.1 table 2.1	104	43	61	51	53	31	12	22	39
7	What diagnostic measures do you carry out during treatment?	text table 4	95	43	52	46	49	31	12	16	36
8	What therapeutic measures do you carry out?	text table 4	93	43	50	47	46	31	12	17	33
9	Which gadget-supported methods have you mastered?	text table 4	95	43	52	46	49	31	12	16	36
10a	How familiar are you with the principle of Castillo-Morales therapy?	table 1.2 table 2.2	104	43	61	51	53	31	12	22	39
10b	I do not know the Castillo-Morales therapy	table 3	4 (104)*	1	3	1	3	1	0	0	3

Question		processed	answers total	ARM 1				ARM 2			
				CET	NCET	EE	LE	CET EE	CET LE	NCET EE	NCET LE
11a	How familiar are you with the principle of nasoalveolar molding (NAM therapy)?	table 1.2<br>table 2.2	104	43	61	51	53	31	12	22	39
11b	I do not know the NAM-Therapie	table 3	72<br>(104)*	29	43	30	42	17	12	15	28
12	In your opinion, for which types of cleft is NAM therapy useful?	excluded	21	-	-	-	-	-	-	-	-
13	When should NAM therapy be administered?	excluded	5	-	-	-	-	-	-	-	-
14a	How familiar are you with the principle of a feeding plate?	table 1.2<br>table 2.2	104	43	61	51	53	31	12	22	39
14b	I do not know feeding plates	table 3	22<br>(104)*	6	16	7	15	3	3	4	12
15	In your opinion, for how long should cleft patients receive speech therapy?	text	64	29	35	31	33	20	9	12	23
16	At what points in time should diagnostic findings be made?	text	49	20	29	26	23	15	5	12	17
17	Which age-dependent findings do you consider to be an indication for speech therapy in cleft patients?	text	99	43	56	48	51	31	12	18	38
18	What should the frequency of speech therapy depend on?	text	97	43	54	47	50	31	12	17	37
19	In which areas do the most frequent problems with cleft patients occur?	text	96	43	53	48	48	31	12	18	35

Question		processed	answers total	ARM 1				ARM 2			
				CET	NCET	EE	LE	CET EE	CET LE	NCET EE	NCET LE
20	In your opinion, which types of clefts develop speech therapy problems more frequently?	excluded	34	-	-	-	-	-	-	-	-
21	How much influence do you think you have as a speech therapist on the success of therapy for patients with cleft?	table 1.3<br>table 2.3	103	43	60	51	52	31	12	21	39
22	How high do you estimate the influence of patient motivation on the success of therapy?	table 1.3<br>table 2.3	103	43	60	51	52	31	12	21	39
23	How high do you estimate the influence of parents on the success of therapy for cleft patients?	table 1.3<br>table 2.3	103	43	60	51	52	31	12	21	39
24	How high do you estimate the influence of the social environment on the therapeutic success of cleft patients?	table 1.3<br>table 2.3	103	43	60	51	52	31	12	21	39
25	How much influence do you think surgeons have on the success of cleft patients' treatment?	table 1.3<br>table 2.3	102	42	60	50	52	30	12	21	39
26	How much influence do you think the width of the cleft has on speech therapy?	table 1.3<br>table 2.3	100	42	58	48	52	30	12	19	39
27	How would you rate the opportunities for *speech development in general* in cleft patients compared to non-cleft patients?	table 1.4<br>table 2.4	102	42	60	49	53	30	12	22	38

Question		processed	answers total	ARM 1				ARM 2			
				CET	NCET	EE	LE	CET EE	CET LE	NCET EE	NCET LE
28	How would you rate the development opportunities in the area of *phonetics* compared to non-cleft patients?	table 1.4<br>table 2.4	104	43	61	51	53	31	12	22	39
29	How would you rate the development opportunities in the area of *phonology* compared to non-cleft patients?	table 1.4<br>table 2.4	104	43	61	51	53	31	12	22	39
30	How would you rate the development opportunities in the area of *speech understanding* compared to non-cleft patients?	table 1.4<br>table 2.4	104	43	61	51	53	31	12	22	39
31	How would you rate the development opportunities in the area of *speech production* compared to non-cleft patients?	table 1.4<br>table 2.4	103	42	61	50	53	30	12	22	39
32	How would you rate the development opportunities in the area of *morphology and syntax* compared to non-cleft patients?	table 1.4<br>table 2.4	102	41	61	50	52	30	11	22	39
33	How would you rate the development opportunities in the area of *orofacial functions* compared to non-cleft patients?	table 1.4<br>table 2.4	103	42	61	50	53	30	12	22	39
34	How do you rate the development opportunities in the area of *voice* compared to non-cleft patients?	table 1.4<br>table 2.4	103	42	61	50	53	30	12	22	39

	Question	processed	answers total	ARM 1				ARM 2			
				CET	NCET	EE	LE	CET EE	CET LE	NCET EE	NCET LE
35	How do you rate the development opportunities in the area of *resonance* compared to non-cleft patients?	table 1.4<br>table 2.4	103	42	61	50	53	30	12	22	39
36	How would you rate the development opportunities in the area of *oral fluency* compared to non-cleft patients?	table 1.4<br>table 2.4	103	42	61	50	53	30	12	22	39
37	What information from the surgical side is important for you as a speech therapist?	text	102	43	59	50	52	31	12	21	38
38	How important is up-to-date information from the surgical side for you as a speech therapist for the success of the therapy?	table 1.5<br>table 2.5	101	41	60	48	53	29	12	21	39
39	What information from the speech therapist do you find useful for the surgeon?	text	100	41	59	49	51	29	12	22	37
40	How important is interdisciplinary collaboration with surgeons to you?	table 1.5<br>table 2.5	103	42	61	50	53	30	12	22	39
41	How important is interdisciplinary collaboration with other specialist disciplines (e.g. ENT, orthodontics) to you?	table 1.5<br>table 2.5	104	43	61	51	53	31	12	22	39
42	How familiar are you with the anatomical malformations of cleft patients?	table 1.6<br>table 2.6	104	43	61	51	53	31	12	22	39
43	How familiar are you with the otolaryngology pathologies?	table 1.6<br>table 2.6	104	43	61	51	53	31	12	22	39
44	In your opinion, with which types of cleft do tympanic effusions occur more frequently?	text	23	12	11	12	11	10	2	3	8

Question		processed	answers total	ARM 1				ARM 2			
				CET	NCET	EE	LE	CET EE	CET LE	NCET EE	NCET LE
45	How familiar are you with the orthodontic pathologies of cleft patients	table 1.6 table 2.6	103	43	60	51	52	31	12	22	38
46	In your opinion, which types of clefts are more likely to cause orthodontic problems?	text	30	16	14	14	16	12	4	3	11
47	How familiar are you with the problem of residual palatal dehiscences after surgical treatment?	table 1.6 table 2.6	102	43	59	49	53	31	12	21	38
48	How much experience do you have with the treatment of patients with residual dehiscences?	table 1.6 table 2.6	102	43	59	49	53	31	12	21	38
49	How important do you consider the health care of patients in an interdisciplinary cleft center for the overall success of treatment?	table 1.5 table 2.5	100	42	58	47	53	30	12	20	38
50	How important do you consider the speech therapy in an interdisciplinary cleft center for patients' development?	table 1.5 table 2.5	99	41	58	46	53	29	12	20	38

*Part b of question: Option to answer "I don't know…" and not give an answer on VAS (part A).
