# Supplementary material for: An evaluation of speech therapy care in the surrounding area of an interdisciplinary cleft lip and palate tertiary care center
Source: Sci Rep. 2025 Feb 18;15:5841. doi: 10.1038/s41598-025-90588-x (PMC11836121; doi:10.1038/s41598-025-90588-x)
Supplement: Supplementary file 2 — Supplementary Information 2. [file 41598_2025_90588_MOESM2_ESM.pdf]

Questionnaire

Speech therapy for cleft lip and palate patients

Age: __________

Sex:

- O Male
- O Female
- O Various

Professional experience in years: __________

Qualification:

- O No degree yet
- O Apprenticeship
- O Bachelor
- O Master
- O Diploma

Further/special qualification: ____

Dear participants,

The following questionnaire is aimed anonymously at speech therapists and their opinions on the subject of cleft lip and palate. Please answer all questions to the best of your knowledge and belief, even if you have no experience with cleft patients or theoretical knowledge. It is only a matter of assessing the facts. The scale markings should be made with vertical lines.
The questionnaire comprises 50 questions on 4 subject areas (general, speech therapy, development opportunities and influences, interdisciplinary cooperation) and will take approximately 12 minutes to complete.
With your participation you can help to improve the therapy of cleft patients!

Thank you very much!

General

1. Do you treat or have you ever treated patients with cleft lip and palate?

   - ○ Yes
   - ○ No

2. If so, how long have you been treating patients with cleft lip and palate?

   - ○ Less than 1 Year
   - ○ 1-5 years
   - ○ 5-10 years
   - ○ Longer than 10 years

3. How many patients with cleft lip and palate have you already treated?
   - ○ Up to 10
   - ○ 10-20
   - ○ 20-50
   - ○ More than 50
   - ○ none

4. How confident do you feel in treating patients with cleft lip and palate?

   0 1 2 3 4 5 6 7 8 9 10

   Not confident — very confident

5. How well educated do you feel in dealing with cleft patients?

   0 1 2 3 4 5 6 7 8 9 10

   Not good — very good

6. How confident do you feel about the expected therapeutic outcome of cleft patients?

   0 1 2 3 4 5 6 7 8 9 10

   Not confident — very confident

Speech therapy

7. What diagnostic measures do you carry out during treatment?

- ○ Phonological-phonetic analysis
- ○ Parent questionnaire
- ○ Orofacial diagnostics
- ○ Cleft lip and palate basic findings
- ○ Independent anamnesis
- ○ Informal questionnaires
- ○ Mirror sample
- ○ Other: ____
- ○ I am not aware of any of the measures listed

8. What therapeutic measures do you carry out?

- ○ Drinking/breastfeeding advice
- ○ Swallowing therapy
- ○ Speech therapy
- ○ Pronunciation therapy
- ○ Other: ____
- ○ I am not aware of any of the measures listed

9. Which gadget-supported methods have you mastered?

- ○ Scape Scope
- ○ Air paddle
- ○ Nasal View
- ○ Bladder exercises
- ○ Suction exercises
- ○ Straw drinking
- ○ Other: ____
- ○ I am not familiar with any of the methods listed

10. How familiar are you with the principle of Castillo-Morales therapy?

0 1 2 3 4 5 6 7 8 9 10

Not familiar very familiar

- ○ I do not know the Castillo-Morales therapy

11. How familiar are you with the principle of nasoalveolar molding (NAM therapy)?

0 — 1 — 2 — 3 — 4 — 5 — 6 — 7 — 8 — 9 — 10

Not familiar ... very familiar

- ○ I do not know the NAM-Therapie → Please go on with Question 14

12. In your opinion, for which types of cleft is NAM therapy useful?

- ○ Cleft lip and palate on both sides
- ○ Cleft lip and palate unilateral
- ○ Cleft lip and alveolus
- ○ Cleft palate
- ○ Submucous cleft palate
- ○ Not specified

13. When should NAM therapy be administered?
Please mark a therapy time slot with start and end.

0 — 1 — 2 — 3 — 4 — 5 — 6 — 7 — 8 — 9 — 10

Birth ... 10. Month

14. How familiar are you with the principle of a feeding plate?

0 — 1 — 2 — 3 — 4 — 5 — 6 — 7 — 8 — 9 — 10

Not familiar ... very familiar

- ○ I do not know the principle of a feeding plate

15. In your opinion, for how long should cleft patients receive speech therapy?
Please mark a therapy time slot with start and end.

0 — 1 — 2 — 3 — 4 — 5 — 6 — 7 — 8 — 9 — 10

Birth ... 20. Years

16. At what points in time should diagnostic findings be made?

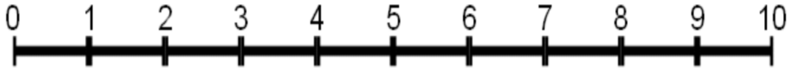

Birth 20. Years

17. Which age-dependent findings do you consider to be an indication for speech therapy in cleft patients?

- ○ Eating/drinking/sucking
- ○ Speech development (basic communication)
- ○ Fine motor skills
- ○ Gross motor skills
- ○ Orofacial status
- ○ Hearing
- ○ Reception
- ○ Expression
- ○ Other: ____

18. What should the frequency of speech therapy depend on?

- ○ Age
- ○ Sex
- ○ Stage of development
- ○ Compliance of the patient
- ○ Compliance of the parents
- ○ Type of cleft
- ○ Cleft width
- ○ Social environment
- ○ Other: ____

19. In which areas do the most frequent problems with cleft patients occur?

- ○ Speech understanding
- ○ Speech production
- ○ Phonetics
- ○ Phonology
- ○ Morphology/syntax
- ○ Orofacial status
- ○ Voice/resonance
- ○ Oral fluency

20. In your opinion, which types of clefts develop speech therapy problems more frequently?
Please rank the cleft types in ascending order from rare (1) to frequent (5).

- ○ Cleft lip and palate on both sides
- ○ Cleft lip and palate unilateral
- ○ Cleft lip and alveolus
- ○ Cleft palate
- ○ Submucous cleft palate
- ○ Not specified

Development opportunities and influences

21. How much influence do you think you have as a speech therapist on the success of therapy for patients with cleft?

0 1 2 3 4 5 6 7 8 9 10

Small extensive

22. How high do you estimate the influence of patient motivation on the success of therapy?

0 1 2 3 4 5 6 7 8 9 10

Small extensive

23. How high do you estimate the influence of parents on the success of therapy for cleft patients?

0 1 2 3 4 5 6 7 8 9 10

Small extensive

24. How high do you estimate the influence of the social environment on the therapeutic success of cleft patients?

0 1 2 3 4 5 6 7 8 9 10

Small extensive

25. How much influence do you think surgeons have on the success of cleft patients' treatment?

0 1 2 3 4 5 6 7 8 9 10

Small extensive

26. How much influence do you think the width of the cleft has on speech therapy?

0 1 2 3 4 5 6 7 8 9 10

Small extensive

<u>Please note the changed division of the scale!</u>

27. How would you rate the opportunities for *speech development in general* in cleft patients compared to non-cleft patients?

0 1 2 3 4 5 6 7 8 9 10

Worse **same** better

28. How would you rate the development opportunities in the area of *phonetics* compared to non-cleft patients?

0 1 2 3 4 5 6 7 8 9 10

Worse **same** better

29. How would you rate the development opportunities in the area of *phonology* compared to non-cleft patients?

0 1 2 3 4 5 6 7 8 9 10

Worse **same** better

30. How would you rate the development opportunities in the area of *speech understanding* compared to non-cleft patients?

0 1 2 3 4 5 6 7 8 9 10

Worse **same** better

31. How would you rate the development opportunities in the area of *speech production* compared to non-cleft patients?

0 1 2 3 4 5 6 7 8 9 10

Worse **same** better

32. How would you rate the development opportunities in the area of *morphology and syntax* compared to non-cleft patients?

0 1 2 3 4 5 6 7 8 9 10

Worse **same** better

33. How would you rate the development opportunities in the area of *orofacial functions* compared to non-cleft patients?

0 1 2 3 4 5 6 7 8 9 10

Worse **same** better

34. How do you rate the development opportunities in the area of *voice* compared to non-cleft patients?

0 1 2 3 4 5 6 7 8 9 10

Worse **same** better

35. How do you rate the development opportunities in the area of *resonance* compared to non-cleft patients?

0 1 2 3 4 5 6 7 8 9 10

Worse **same** better

36. How would you rate the development opportunities in the area of *oral fluency* compared to non-cleft patients?

0 1 2 3 4 5 6 7 8 9 10

Worse **same** better

Interdisciplinary Collaboration

37. What information from the surgical side is important for you as a speech therapist?
    - ○ Cleft type: open vs. covered/submucosal
    - ○ Exact localization of the cleft
    - ○ Width of cleft
    - ○ Operation techniques
    - ○ Timing of surgery
    - ○ Non-surgical therapy
    - ○ Assessment of the surgical options
    - ○ Presence/size of the residual palatal holes
    - ○ Morphology of the soft palate
    - ○ Other: ____
    - ○ None

38. How important is up-to-date information from the surgical side for you as a speech therapist for the success of the therapy?

    0 1 2 3 4 5 6 7 8 9 10

    Not important ... very important

39. What information from the speech therapist do you find useful for the surgeon?
    - ○ Speech understanding
    - ○ Speech production
    - ○ Phonetics
    - ○ Phonology
    - ○ Morphology/Syntax
    - ○ Orofacial status
    - ○ Voice/Resonance
    - ○ Oral fluency
    - ○ None

40. How important is interdisciplinary collaboration with surgeons to you?

    0 1 2 3 4 5 6 7 8 9 10

    Not important ... very important

41. How important is interdisciplinary collaboration with other specialist disciplines (e.g. ENT, orthodontics) to you?

0 1 2 3 4 5 6 7 8 9 10

Not important very important

42. How familiar are you with the anatomical malformations of cleft patients?

0 1 2 3 4 5 6 7 8 9 10

Not familiar very familiar

43. How familiar are you with the otolaryngology pathologies?

0 1 2 3 4 5 6 7 8 9 10

Not familiar very familiar

44. In your opinion, with which types of cleft do tympanic effusions occur more frequently?
Please rank the cleft types in ascending order from rare (1) to frequent (5).

- ○ Cleft lip and palate on both sides
- ○ Cleft lip and palate unilateral
- ○ Cleft lip and alveolus
- ○ Cleft palate
- ○ Submucous cleft palate
- ○ Not specified

45. How familiar are you with the orthodontic pathologies of cleft patients?

0 1 2 3 4 5 6 7 8 9 10

Not familiar very familiar

46. In your opinion, which types of clefts are more likely to cause orthodontic problems?
Please rank the cleft types in ascending order from rare (1) to frequent (5).

- ○ Cleft lip and palate on both sides
- ○ Cleft lip and palate unilateral
- ○ Cleft lip and alveolus
- ○ Cleft palate
- ○ Submucous cleft palate
- ○ Not specified

47. How familiar are you with the problem of residual palatal dehiscences after surgical treatment?

0 1 2 3 4 5 6 7 8 9 10

Not familiar very familiar

48. How much experience do you have with the treatment of patients with residual dehiscences?

0 1 2 3 4 5 6 7 8 9 10

None much

49. How important do you consider the health care of patients in an interdisciplinary cleft center for the overall success of treatment?

0 1 2 3 4 5 6 7 8 9 10

Not important very important

50. How important do you consider the speech therapy in an interdisciplinary cleft center for patients' development?

0 1 2 3 4 5 6 7 8 9 10

Not important very important

51. Further comments:

____
____
____
____
____

Thank you for your participation!
Please send the questionnaire back to us within the next 4 weeks in the stamped envelope!

Alternatively, you can also scan the questionnaire and send it to the following email address:

kaschaffrath@ukaachen.de

We will be happy to send you the results after evaluation. Please enter your e-mail address if you are interested:

____
